# Evaluation of the impact of two citrus plants on the variation of *Panonychus citri* (Acari: Tetranychidae) and beneficial phytoseiid mites

**DOI:** 10.1515/biol-2022-0837

**Published:** 2024-03-16

**Authors:** Amine Assouguem, Abdelmalek Boutaleb Joutei, Rachid Lahlali, Mohammed Kara, Ahmed Bari, Essam A. Ali, Hafize Fidan, Hajar Zine Laabidine, Younouss El Ouati, Abdellah Farah, Abderrahim Lazraq

**Affiliations:** Laboratory of Functional Ecology and Environment, Faculty of Sciences and Technology, Sidi Mohamed Ben Abdellah University, Imouzzer Street, P.O. Box 2202, Fez, Morocco; Department of Protection of Plants and Environment, National School of Agriculture, Meknes, Morocco; Laboratory of Biotechnology, Conservation and Valorisation of Natural Resources (LBCVNR), Faculty of Sciences Dhar El Mehraz, Sidi Mohamed Ben Abdallah University, BP 1796 Atlas, Fez 30000, Morocco; Department of Pharmaceutical Chemistry, College of Pharmacy, King Saud University, Riyadh, Saudi Arabia; Department of Tourism and Culinary Management, Faculty of Economics, University of Food Technologies, Plovdiv, Bulgaria; Laboratory of Biotechnology, the Law, Philosophy and Society Laboratory (ESSOR), The Faculty of Law, Economic and Social Sciences, Sidi Mohamed Ben Abdallah University, BP 1796 Atlas, Fez 30000, Morocco; Laboratory of Applied Organic Chemistry, Faculty of Sciences and Technology, Sidi Mohamed Ben Abdellah University, Imouzzer Street, Fez P.O. Box 2202, Morocco

**Keywords:** *Panonychus citri*, citrus varieties, monitoring, predator, pest

## Abstract

The abundance of *Panonychus citri* McGregor 1916 (Acari: Tetranychidae) and its associated enemies (*Euseius stipulatus* Athias-Henriot, 1960; *Typhlodromus* sp.; *Phytoseiulus persimilis* Athias-Henriot, 1957) was studied on two 12-year-old citrus cultivars, specifically Clementine “Nules” (*Citrus Clementina*) and Valencia (*Citrus sinensis*), in the Gharb region of Morocco. Throughout the entire monitoring period in the Valencia late cultivar, the density of the spider mite *P. citri* on leaves was notably higher at 38.0% (*n* = 1,212 mobile forms). Predator *P. persimilis* exhibited a leaf occupancy of 25.0% (*n* = 812), followed by *Typhlodromus* sp. at 20.0% (*n* = 643). Conversely, the abundance of *E. stipulatus* was lower at 17.0% (*n* = 538). In the Nules variety, *P. citri* abundance recorded a higher percentage at 48.0% (*n* = 1,922). *E. stipulatus* emerged as the most abundant predator at 23.0% (*n* = 898), followed by *P. persimilis* with 16.0% (*n* = 639). Meanwhile, the population of *Typlodromus* sp. remained notably low at 13.0% (*n* = 498). Regarding the fluctuation of the different mites studied on the two cultivars across monitoring dates, the period from May 4 to June 1 was characterized by low temperatures and a diminished presence of mite populations (*P. citri, E. stipulatus, Typhlodromus* sp., and *P. persimilis*). However, from June 7 to June 19, characterized by high temperatures, a notable increase in the presence of mite populations was observed. As regards the effect of the variety on the different mites studied, the varietal impact was significant.

## 1 Introduction

Citrus is one of the most important fruit crops worldwide [1]. It belongs to the Rutaceae family with 140 genera and 1,300 species, including fundamental groups like orange, lemons, mandarin, and pummelos. Citrus fruits originate from tropical and subtropical areas of Asia and Oceania [2]. The total worldwide production of citrus is 143,70 million tons [3]. The Moroccan citrus production holds the fifth position in the Mediterranean with an average of 1,725,000 tons per year, including 815,000 tons of orange and 910,000 tons of mandarins [4]. The citrus industry is very important for the socio-economic development of the country since it generates an annual turnover of 3 billion dirhams and nearly 21 million working days [5].

The national citrus orchard predominantly comprises three major cultivars: Valencia-Late (constituting 27%), Clementine, including Nules (comprising 26%), and Navel (accounting for 17%) of the total citrus production (Yacoubi [6]). Valencia late (Maroc late) is a large, vigorous tree native to Morocco. It is considered the latest variety of all oranges, ripening from mid-March to July [7]. Nules were discovered in the city of Nules in Castellon, and they are very popular in Spain [8]. This fruit is of a large size, with grainy skin and juicy flesh, available from October to December [9].

*Panonychus citri* McGregor 1916 (Acari: Tetranychidae) is a primary pest of citrus and causes considerable damage [10,11]. Classified within the Tetranychidae family of the Acari order, this diminutive arachnid has gained notoriety for the substantial damage it inflicts upon citrus crops, making it a focal point for comprehensive pest management strategies [12].

Emerging from McGregor’s initial classification in 1916, *P. citri* has evolved into a recognized and pervasive threat to citrus orchards worldwide [5,13]. The mite’s distinctive modus operandi involves a pronounced preference for feeding on the lower surface of citrus leaves, extracting the contents of mesophyll cells. This feeding behavior induces a weakening of the plant, resulting in a subsequent decline in both the quality and yield of citrus fruits [14].

A significant challenge in controlling *P. citri* lies in its remarkable reproductive capacity under favorable conditions, amplifying the potential for extensive crop damage [15]. Consequently, the implementation of effective management strategies becomes imperative for preserving citrus crops and fostering sustainable agricultural practices [5,16]. Various approaches have been deployed to address the menace posed by the citrus red mite. A natural and environmentally friendly method involves the introduction of predatory mites, such as *P. persimilis*, *Typhlodromus* sp., and *Euseius stipulatus* [5]. These natural predators act as a biological and sustainable solution, keeping *P. citri* populations in check by preying on them [17,18].

*E. stipulatus*, *P. persimilis,* and *Typhlodromus* sp. were observed feeding on *P. citri* in different Moroccan regions: Kenitra, Sidi Slimane, Belksiri, Sidi Kacem, Tazi, Sidi abdelaaziz, and Rabat [12,19]. Nevertheless, the prevalent use of synthetic insecticides remains a common practice in controlling citrus pests, including *P. citri.* While effective in certain instances, this approach presents its own set of challenges [20,21]. Synthetic insecticides can prove toxic to beneficial predatory mites, disrupting the delicate ecological balance and diminishing the overall efficacy of biological control methods [5]. *Panonychus citri* emerges as a formidable adversary to citrus crops, necessitating a nuanced and multifaceted approach to pest management. Striking a balance between deploying natural predators, like predatory mites, and employing synthetic insecticides judiciously and sustainably is paramount in mitigating the impact of this pest on citrus orchards [22]. Such an approach ensures the continued health and productivity of citrus crops while minimizing environmental repercussions. The density, fecundity, and outbreak potential of *P. citri* are intricately woven into a complex tapestry of environmental factors, encompassing temperature and humidity dynamics, along with the nuanced interplay of seasonal variations and the unique characteristics of host plant varieties. A profound understanding of these multifaceted interactions is indispensable for the development of targeted and effective strategies aimed at managing and mitigating the impact of *P. citri* infestations in agricultural settings. This study will provide important data on the fluctuation of mites over time in citrus cultivars without the use of pesticides. This is of great importance for future comparative research to determine the fluctuation of mite biodiversity, infestation rates, and spread rate of pests according to variety category. The goal of this study is to evaluate the varietal impact of two citrus varieties (Nules [Mandarin] and Valencia [Orange]) on fluctuations of *P. citri* and beneficial phytoseiid mites (*E. stipulatus*, *Typhlodromus* sp., and *P. persimilis*). Additionally, the influence of temperature, relative humidity (RH) and variety on the abundance of *P. citri* and predators (*P. persimilis Typhlodromus* sp. and *E. stipulatus*) was also determined.

## 2 Materials and methods

### 2.1 Study area

**Figure 1:**
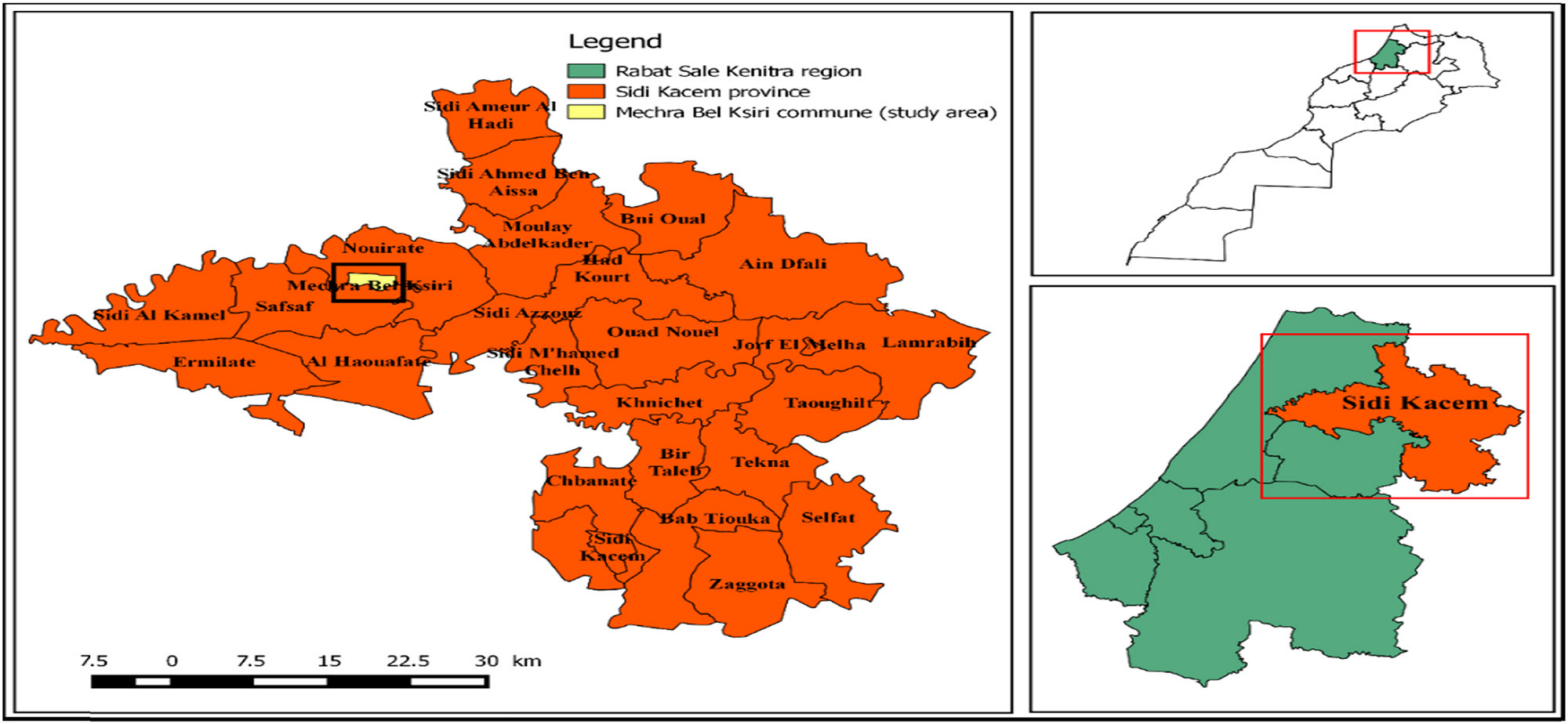
The location of citrus orchards studied [5].

This work was performed in Mechra Bel Ksiri area (Figure 1), which is located on the north of Oued Sebou at an altitude of about 300–500 m above sea level in the Gharb region [23]. This region is well known for the production of citrus, cereals, and vegetable crops due to the adequate characteristics of climate and soil [24]. The Gharb area is characterized by a Mediterranean climate with annual precipitation varying between 480 and 600 mm/year, and an average temperature of 27°C in summer and 13°C in winter [25].

### 2.2 Sampling design

Two plots (4 ha each) planted with two different citrus cultivars were randomly selected for this survey; one was planted with Nules variety (*Citrus Clementina*) and second was planted with Valencia late variety (*Citrus sinensis*). The activity of the pest (*P. citri*) and its predators (*E. stipulatus, Typhlodromus* sp., and *P. persimilis*) was monitored weekly.

A block of ten trees was selected and monitored weekly. Ten leaves were collected from each tree, resulting in a total of 100 leaves per plot. The leaves were gathered from different directions (North, East, South, and West) and at various heights of the tree, ranging from 1.5 to 3.5 m. Ten replicates were performed independently [26,27]. The total number of predators and phytophagous mite *P. citri* found on the ten leaves of each plot was recorded separately [28]. The different mobile forms of the mites studied on each leaf were determined and counted on both surfaces of the leaf with a professional eye loupe 10×. The inspections were conducted on a weekly basis from April 12 to June 1. Temperature and RH data were meticulously recorded in the field utilizing the Davis Instruments Vantage Pro2.

## 3 Statistics

The results were expressed as percentage and mean ± SD. In addition, to evaluate the density of each species of the studied mites, we calculated the average of mobile forms found of *P. citri* and their predators (*P. persimilis*, *Typhlodromus* sp., and *E. stipulatus*) in both plots [25]. To assess the impact of citrus cultivars, monitoring dates, and their interactions on mite fluctuation, a general linear model (GLM) was performed, and mean values were separated with Tukey HSD post hoc test at *p* < 0.05 [5]. We checked the normality and homogeneity of variance for all variables with the Kolmogorov–Smirnov test. These analyses were performed using Minitab.

To identify the correlation between variables (weeks and temperature) and the mite populations studied, a principal component analysis was applied. Hierarchical cluster analysis (HCA) was used to better visualize different groups. Principal component analysis, HCA, and multiple linear regression were carried out using JMP Pro 14 software (SAS company, Cary, Carolina, USA).

## 4 Results

### 4.1 Density rate of the studied mites

**Figure 2:**
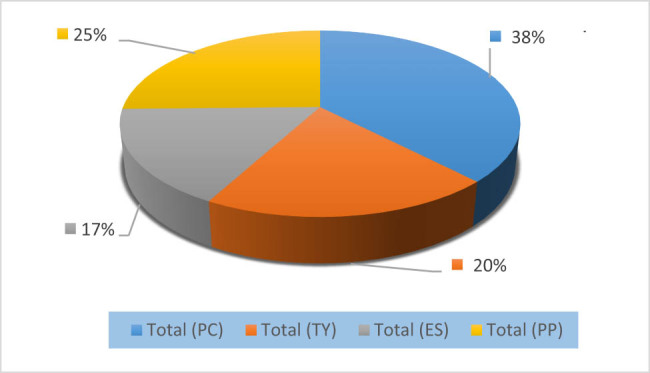
The population of the phytophagous mite *P. citri* (PC) and its enemies *E. stipulatus* (ES), *Typhlodromus* sp. (TY), and *P. persimilis* (PP) during the entire follow-up period on the Valencia late variety.

On the variety Valencia late (Figure 2), during the whole monitoring period, leaf density by *P. citri* was higher by 38% with mobile forms *n* = 1,212. The predator *P. persimilis* presented the most important percentage with 25% (*n* = 812). The proportion of *Typhlodromus* sp. was 20% (*n* = 643), and the abundance of *E. stipulatus* was lower (17%, *n* = 538).

**Figure 3:**
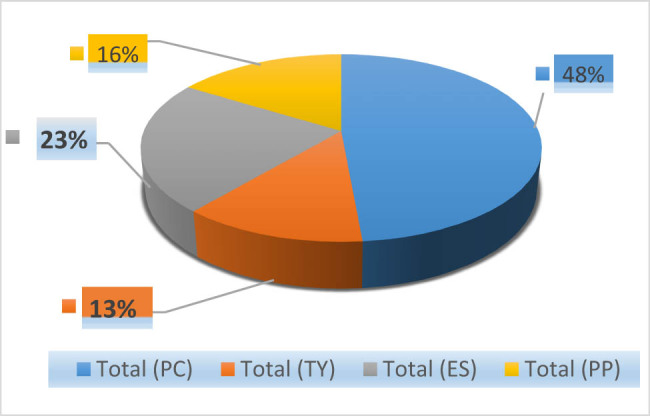
The population of the phytophagous mite *P. citri* (PC) and its enemies *E. stipulatus* (ES), *Typhlodromus* sp. (TY), and *P. persimilis* (PP) during the entire follow-up period on the Nules variety.

On the variety Nules, the density of *P. citri* was 48% (*n* = 1,922). *E. stipulatus* was the most abundant predator species, with a proportion of 23% (*n* = 898), followed by *P. persimilis* with 16% (*n* = 639). While the rate of the predator *Typlodromus* sp. was very low (13%, *n* = 498) (Figure 3).

### 4.2 Varietal impact on mite populations

Tables 1 and 2 present the influence of a variety of dates and their interactions on the averages of different mites studied. Nules (V2) and Valencia late (V1) cultivars showed a significant impact in the abundance of the pest *P. citri* with totally different averages, 15.15A ± 2.34 per tree on Valencia late and 24.02B ± 2.84 per tree on Nules. While on the predators, we noticed that the varietal impact on *E. stipulatus* was significant. On Valencia late, the average was 06.72A ± 1.55 per tree; on Nules, it was almost double 11.22B ± 2.01 per tree. The same result was observed on *Pytoseiulus persimilis* with significantly different averages: 10.15A ± 1.80 on Valencia late and 7.98B ± 1.30 on Nules. In contrast, the two cultivars also show an impact on *Typhlodromus* sp. with relatively different means: 08.04B ± 1.55 per tree on Valencia late and 06.70A ± 1.02 per tree on Nules variety. At the same time, we note that the interaction between dates × variety has a significant impact on the change in the studied mites, except for *P. persimilis.*

**Table 1:** Variance analysis of different mite densities using GLM according to dates, variety, and its interactions (M: monitoring)

Species	Source	DF	Adj SS	Adj MS	*F*-Value	*P*-Value
*P. citri*	Variety	1	3,151	3150.63	213.84	0.000*
	Monitoring dates (weeks)	7	35,696	5099.45	346.12	0.000*
	M. dates*variety	7	2,550	321.48	21.82	0.000*
*Typhlodromus* sp.	Variety	1	71.56	71.56	17.09	0.000*
	Monitoring dates (weeks)	7	1808.89	258.41	61.72	0.000*
	M. dates*variety	7	211.89	30.271	7.23	0.000*
*E. stipulatus*	Variety	1	810	810	27.90	0.000*
	Monitoring dates (weeks)	7	6247.2	892.46	30.74	0.000*
	M. dates*variety	7	1184.5	169.21	5.83	0.000*
	Variety	1	187.06	187.06	24.15	0.000*
*P. persimilis*	Monitoring dates (weeks)	7	4477.19	639.60	82.57	0.000*
	M. dates*variety	7	40.49	5.78	0.75	0.633

^*^Statistically significant *p*-values (*p* < 0.05).

**Table 2:** Average population size of the different species studied (PC: *P. citri*; ES: *E. stipulatus*; TY: *Typhlodromus* sp.; PP: *P. persimilis*) depending on the two varieties (V1: Valencia late variety; V2: Nules variety)

	*P. citri*	*Typhlodromus* sp.	*E. stipulatus*	*P. persimilis*
Valencia late	15.15A ± 2.34	06.72A ± 1.55	08.04A ± 1.55	10.15A ± 1.80
Nules	24.02B ± 2.84	11.22B ± 2.01	06.70B ± 1.02	7.98B ± 1.30

Mean values that do not share a letter are significantly different (*p* < 0.05).

Concerning the comparisons between the average level of mite population per tree on the two cultivars, the levels of *P. citri* and *E. stipulatus* were high on Nules compared to Valencia late, while the proportion of *P. persimilis* and *Typhlodromus* sp. was high on Valencia late (Table 2).

On the variety Valencia late, the results showed that the average population of *P. citri* remained consistently low from the first week to the fourth week. The recorded averages ranged between 4.20C ± 1.44 and 5.70C ± 1.33 mobile forms per tree (Table 2). These observations were made during a specific temperature range of 28–32°C and RH between 20 and 27% (Table 3). In the 5th week, as the temperature increased to 34°C and RH 20%, the average *P. citri* increased to 14.90B ± 2.22. Peak averages were documented during the 6th to 8th week, coinciding with a temperature range of 37–39°C and RH levels spanning from 15% to 31%. Specifically, the recorded values were 26.50A ± 3.12 in the 6th week, 29.80A ± 3.94 in the 7th week, and 31.30A ± 3.81 in the 8th week. Likewise, the predator *E. stipulatus* exhibited a noteworthy surge from the initial to the final week of observation, escalating from 1.90D ± 1.00 to 12.30A ± 2.14. The rise in temperature, concurrent with the increase in *P. citri* population and RH, facilitated a significant expansion of the *Typhlodromus* sp. predator, surging from 2.50E ± 0.44 in week 1 to 14.10A ± 2.97 in week 8. Furthermore, the average counts of *P. persimilis* experienced a substantial increase from 2.40D ± 0.66 in week 1 to 18.50A ± 2.80 in the concluding week of monitoring.

**Table 3:** Mean values of different species studied (PC: *P. citri*; ES: *E. stipulatus*; TY: *Typhlodromus* sp.; PP: *P. persimilis*) according to the different follow-up dates on Valencia late variety

	PC	TY	ES	PP
May 4	4.90C ± 1.23	2.50E ± 0.44	1.90D ± 1.00	2.40D ± 0.66
May 11	4.20C ± 1.44	3.30DE ± 1.01	2.20D ± 1.21	3.80CD ± 1.11
May 18	3.90C ± 1.34	5.40CD ± 1.25	3.80CD ± 1.22	6.70BCD ± 1.31
May 25	5.70C ± 1.33	06.50C ± 1.67	6.60B ± 1.34	8.30BC ± 1.47
June 1	14.90B ± 2.22	07.70C ± 1.42	5.20BC ± 1.32	9.40B ± 2.10
June 7	26.50A ± 3.12	11.20B ± 2.55	10.50A ± 2.01	15.50A ± 2.31
June 12	29.80A ± 3.94	13.60AB ± 2.49	11.30A ± 2.43	16.60A ± 2.43
June 19	31.30A ± 3.81	14.10A ± 2.97	12.30A ± 2.14	18.50A ± 2.80

Mean values in the same column with different letters are significantly different (*p* < 0.05).

On the Nules variety, *P. citri, E. stipulatus, P. persimilis*, and *Typhlodromus* sp. exhibited relative stability in the initial 4 weeks, followed by a significant increase from the 5th week onwards. The average count of *P. citri* notably escalated from 4.30G ± 1.01 in week 1 to 53.20A ± 3.11 in week 8. Concurrently, *Typhlodromus* sp., *E. stipulatus*, and *P. persimilis* demonstrated substantial increases from week 1 (28°C, RH 25%) recording 4.30C ± 1.03, 2.40B ± 1.00, and 2.00F ± 1.08, respectively to the concluding week (week 8; 39°C, RH 31%), reaching 9.30A ± 1.56, 25.50A ± 2.88, and 16.30A ± 2.07, respectively (Table 4).

**Table 4:** Temperatures and Relative humidity recorded during the monitoring period

Follow-up date	04 May	11 May	18 May	25 May	1 June	7 June	12 June	19 June
Temperature (°C)	28	27	30	32	34	38	37	39
Relative humidity (%)	25	20	25	27	20	15	22	31

**Figure 4:**
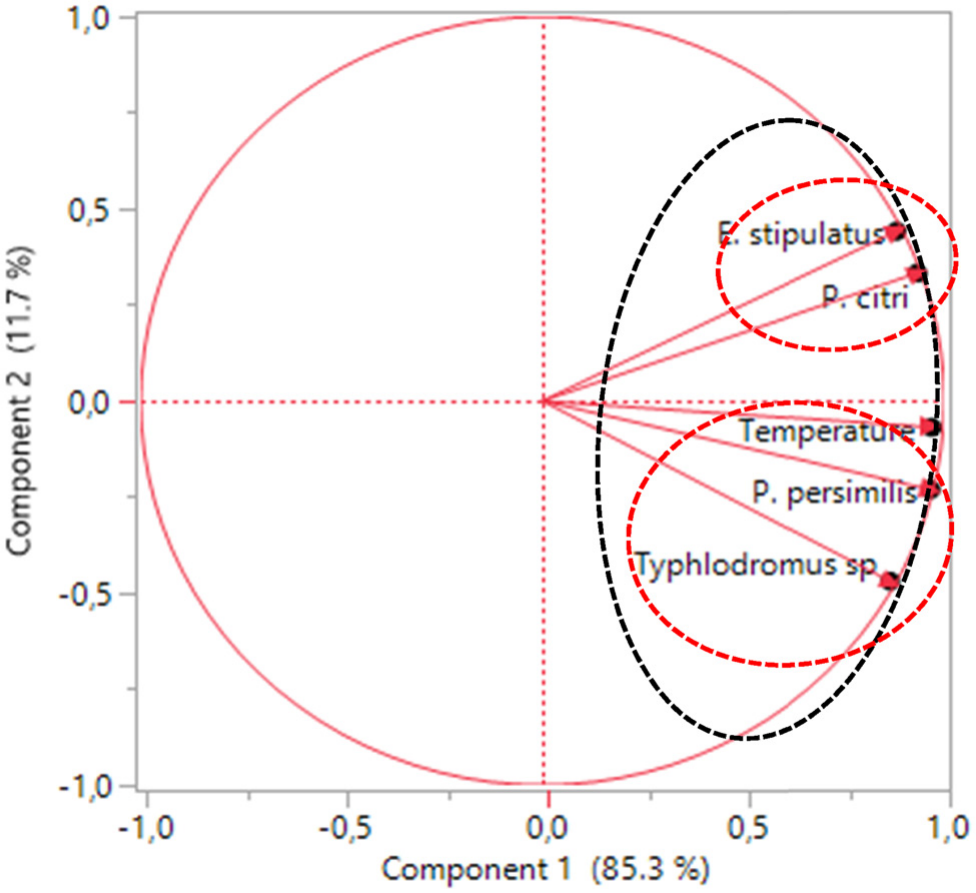
Loading plot represents the projection of the variables on PC1 and PC2. PC: principal component.

Principal component analysis was employed to identify correlations between the variables studied (T° and Follow-up weeks) in a graphical manner and mite populations (*P. citri, Typhlodromus* sp., *E. stipulatus*, and *P. persimilis*). The calculation of the Kaiser–Meyer–Olkin (KMO) index and the Bartlett test revealed that the principal component analysis is feasible in our study since the KMO index (0.76) is greater than 0.5 and the Bartlett test is highly significant (*p* < 0.05). Figure 4 and Table 5 revealed some correlation between the variables studied. A strong positive correlation (>0.7) between (a) temperature and the four mites studied (*P. citri, Typhlodromus* sp., *E. stipulatus*, and *P. persimilis*), (b) *P. citri* and (*E. stipulatus* and *P. persimilis*), (c) *Typhlodromus* sp. and *P. persimilis,* and (d) *E. stipulates* and *P. persimilis*. This means that when the temperature increase the density of the mite population (*P. citri, E. stipulatus*, *Typhlodromus* sp., and *P. persimilis*) increases, and when the presence of *P. citri* is high, the presence of beneficial phytoseiid mites was important.

**Table 5:** Mean values of different species studied (PC: *P. citri*; ES: *E. stipulatus*; TY: *Typhlodromus* sp.; PP: *P. persimilis*) according to the different follow-up dates on Nules variety

	PC	TY	ES	PP
May 4	4.30G ± 1.01	4.30C ± 1.03	2.40B ± 1.00	2.00F ± 1.08
May 11	7.00FG ± 1.32	4.50C ± 1.12	2.60B ± 1.04	2.60EF ± 1.13
May 18	8.40EF ± 1.01	5.10C ± 1.48	4.20B ± 1.44	4.60DE ± 1.29
May 25	12.20E ± 2.03	5.40C ± 1.34	8.20B ± 1.54	5.50CD ± 1.37
June 1	20.20D ± 2.33	6.00BC ± 1.24	9.40B ± 1.11	7.70C ± 1.55
June 7	37.70C ± 3.12	6.20BC ± 1.18	12.00B ± 2.44	11.80B ± 1.32
June 12	49.20B ± 3.33	9.00AB ± 1.43	25.50A ± 2.79	13.40B ± 1.77
June 19	53.20A ± 3.11	9.30A ± 1.56	25.50A ± 2.88	16.30A ± 2.07

Mean values in the same column with different letters are significantly different (*p* < 0.05).

The loading plot indicated that all variables are well represented by the first principal component. The first component explains 85.30% of data variability and the second component explains 11. 70% of the variables. Therefore, these two main components can explain 97% of the total information (Figure 4).

**Figure 5:**
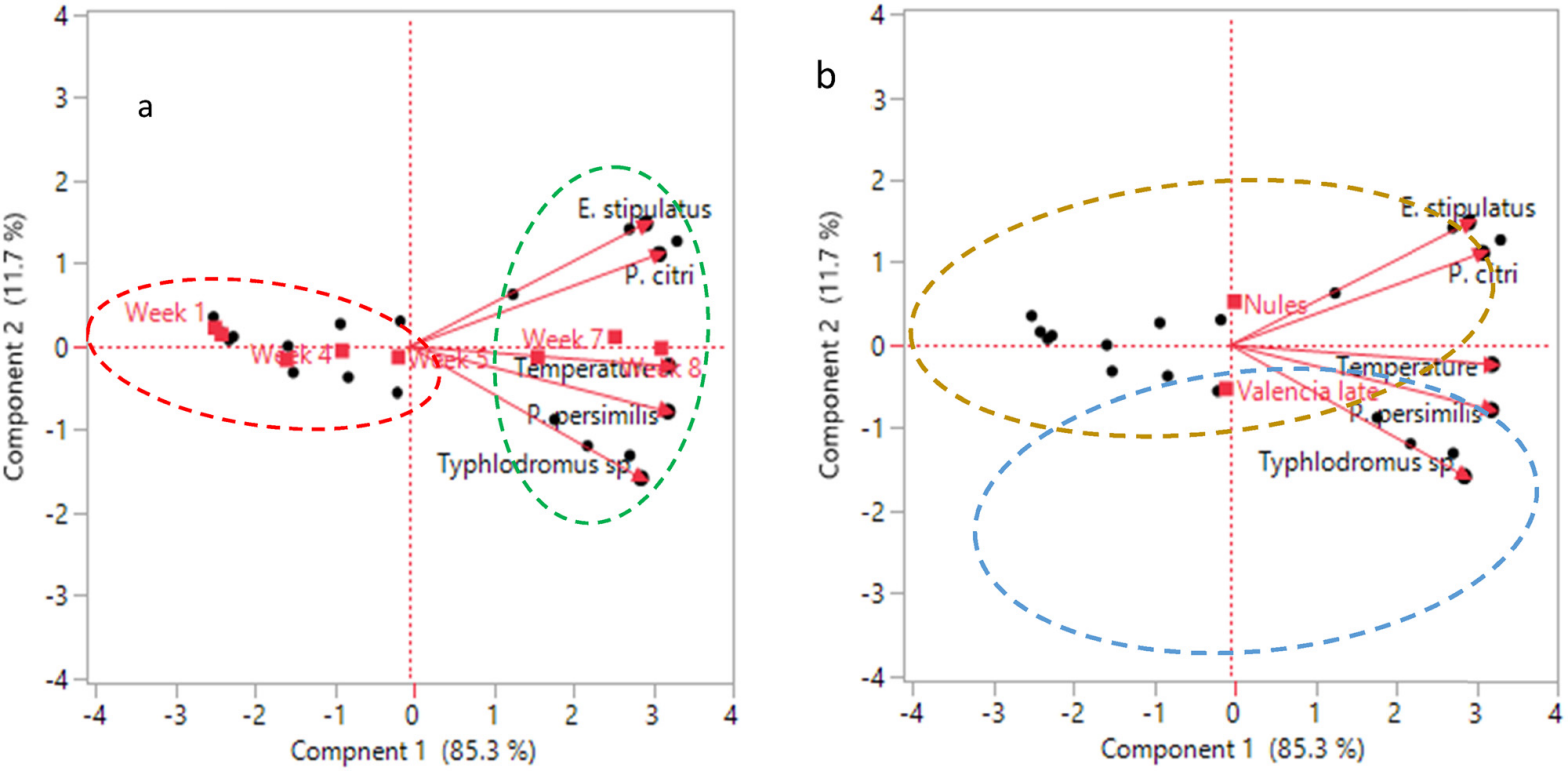
Biplot represents the projection of the individuals and variables on the PC1 and PC2. (a) Projection of the variables according to the weeks. (b) Projection of the variables according to the variety.

The biplot (Figure 5(a)) demonstrates that the weeks are divided into two main groups, one containing the weeks 1–5 formed a group on the left (red circle), and the second from weeks 6 to 8 formed a group on the right (green circle). The graph also reveals that the group with the red circle contained the lowest temperature and a low density of the mite populations (*P. citri*, *E. stipulatus*, *Typhlodromus* sp., and *P. persimilis*), especially in the individuals corresponding to week 1 and week 2, while the other group with the green circle was dominated by the highest temperature and a high presence of the mite populations, especially in the individuals corresponding to week 8. Figure 5(b) indicates that the Nules variety is characterized by a high presence of *P. citri, E. stipulatus*, while the valencia late variety is marked by a high presence of *Typhlodromus* sp. and *P. persimilis*.

**Figure 6:**
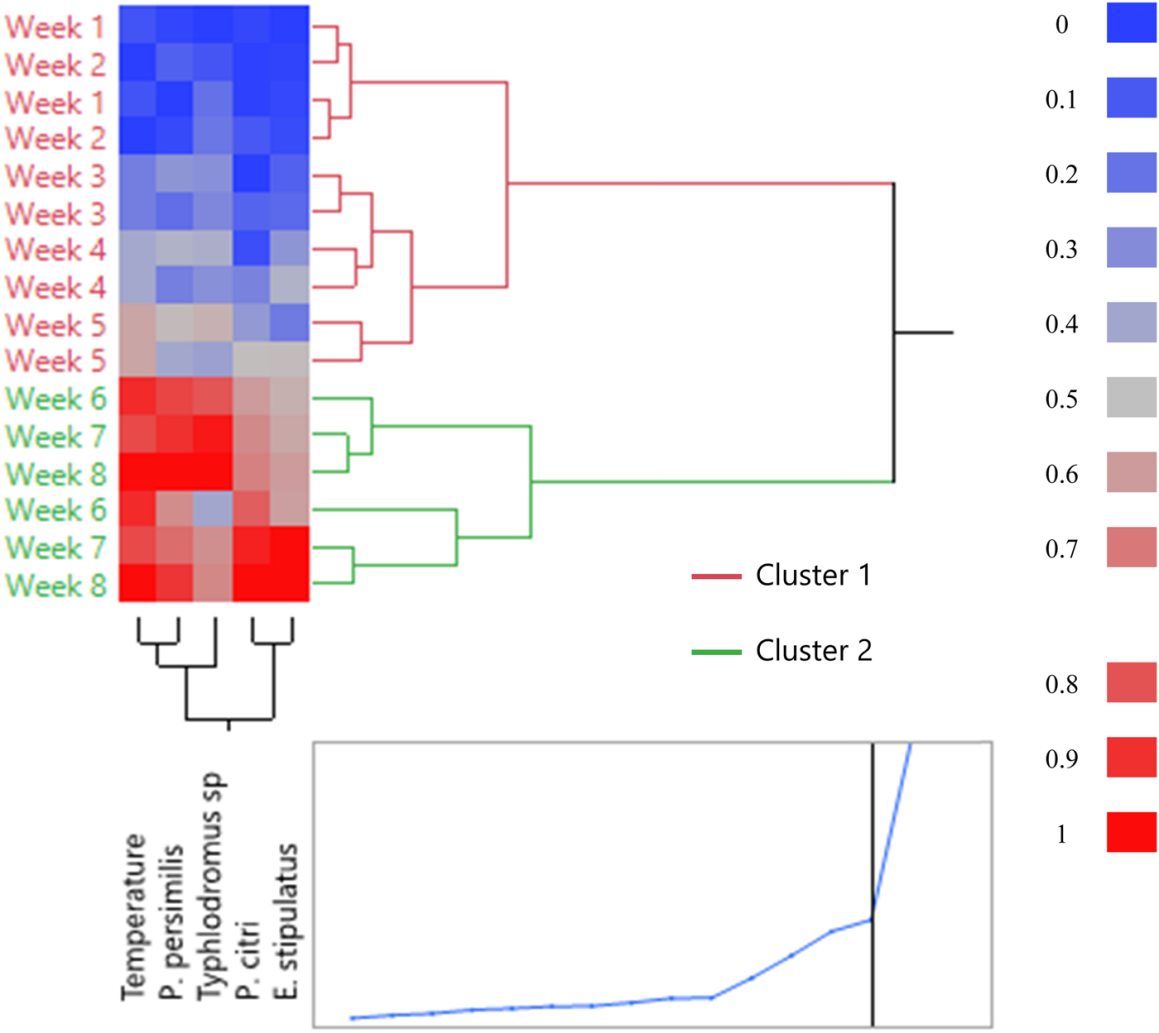
HCA of Valencia late and Nules variety samples according to the variables studied by applying the Euclidean distance between clusters method.

In order to better visualize the classification of the studied populations according to temperature and monitoring dates, an HCA was performed (Figure 6). As the results found previously by the projection of individuals on PC1 and PC2 in the PCA analysis. Cluster 1 represents the group from week 1 to week 5, and Cluster 2 represents the group from week 6 to week 8. The first cluster (weeks 1–5) is marked by a low temperature and a low presence of the mite populations (*P. citri, E. stipulatus*, *Typhlodromus* sp., and *P. persimilis*) (blue color). However, the second cluster (weeks 6–8) is defined by a high temperature and a high presence of the mite populations (red color) (Figure 6).

## 5 Discussion

Our nationwide survey represents the first research focused on studying the fluctuation of *P. citri* and beneficial phytoseiid mites in relation to two citrus cultivars (Valencia late and Nules). Furthermore, both nationally and internationally, species within the beneficial phytoseiid mite family are at risk of extinction. In this context, our study aims to follow up the activity of *P. citri* and their enemies (*E. stipulatus, Typhlodromus* sp., and *P. persimilis*) on two citrus varieties (Nules and Valencia late) without chemical intervention. In light of this broader conservation concern, the study adopts a unique approach by refraining from chemical intervention. This decision enables an exploration of the natural dynamics between *P. citri* and its natural enemies, including *E. stipulatus*, *Typhlodromus* sp., and *P. persimilis*, on the specified citrus varieties. The research aims to provide valuable insights into the interplay between pests and predators without external interference, contributing not only to the field of pest management but also to the broader context of biodiversity conservation. This study aligns with the growing emphasis on sustainable and environmentally friendly agricultural practices, fostering a holistic understanding of ecosystem dynamics in citrus cultivation.

Over the 2-month monitoring period, we noticed that the number of *P. citri* was much higher than their predators (*E. stipulatus, Typhlodromus* sp., and *P. persimilis*). Various studies conducted under different circumstances demonstrate that *P. citri* is a major pest of various crops worldwide and exceeds the capacity of these predators by its high resistance to environmental constraints [21,30]. Damage to host plants by this mite, which feeds on leaves, twigs, and fruit, results in severe defoliation and the pale appearance of immature fruit, results in severe defoliation and pale appearance of immature fruit, affecting citrus quantity and quality [14,15,31]. On the other hand, the abundance of beneficial phytoseiid mites (*E. stipulatus*, *Typhlodromus* sp., and *P. persimilis*) on both cultivars (Valencia late and Nules) exceeds that of *P. citri*, the sum of the percentages of these three predators was 62% on Valencia late and 52% on the Nules variety. Based on the results of different research, the control by these three predators studied can regulate the rate of this pest. In Japan, the utilization of natural enemies in a rational, integrated control method allowed for effective control of *P. citri* and several spider mites [22,32,33]. In another study, *Typhlodromus occidentalis* Nesbitt, 1951, and *E. stipulatus* exerted the greatest control on *P. citri* and *Tetranychus urticae* [34]. Furthermore, in Eastern Spain’s citrus orchards, the use of generalist and specialist phytoseiid predators are complementary and can together reduce the spatial, temporal, and developmental refugia of phytophagous citrus mites, such as *P. citri and T. urticae,* which remain an unsolved problem [35,36,37].

Our results show that the two studied cultivars have a significant impact on the abundance of *P. citri* and on the two predators, *E. stipulatus* and *P. persimilis*. Similar findings were reported by Amin et al. [38], indicating that the variety can influence the fluctuation of pests and predators. Another study was performed in the open field on five cotton cultivars to compare the infestation degree by the pests *Aphis gossypii* Glover, 1877, (Hemiptera: Aphididae) and *Amrasca devastans* Dist. (Hemiptera: Cicadellidae). The results showed that the type of variety could influence the infestation level (Amin et al. [39]). Generally, the type of vegetation and the category of the variety can influence the abundance and composition of Phytoseiidae [40,41].

Our results show that the rate of *P. citri* increased significantly from the first week (May 4) to the last week of monitoring (June 19); this is due to the increase in temperature from 28 to 39°C. The same results were obtained on clementine in the region of Gharb (Morocco) without the practice of acaricide treatments; in order to know the severity of the outbreak and the rate of *T. urticae*, this study showed that the degree of infestation of the trees was 10% in mid-April and increased rapidly to 87% in July [42]. The study by Tello Mercado et al. also demonstrated that the increase in temperature plays a major role in the fluctuation of mite populations [43]. Other studies confirmed that temperature is the most important environmental factor affecting insect population dynamics [44,45].

## 6 Conclusions

The provided conclusion encapsulates significant insights derived from the study and outlines crucial considerations for future research in the realm of mite-plant interactions. Here’s an expansion on the key points: (1) The first key finding underscores the influence of citrus variety on the fluctuation and abundance of both pests (such as *Panonychus citri*) and their predators. This implies that the choice of citrus cultivar can have a discernible impact on the ecological balance between mites and their natural enemies. (2) The second key finding emphasizes the predominant role of temperature and RH in influencing the level of infestation and the rate of propagation of mites. This highlights the sensitivity of mite populations to climatic conditions, particularly temperature fluctuations. Recognizing the significance of temperature in mite dynamics is crucial for predicting and managing infestations. The conclusion underscores the importance of future studies in advancing our understanding of mite–plant interactions. These studies are envisioned as effective tools for comprehending the changing fluctuation patterns of mites. Importantly, the suggested future investigations should incorporate a holistic approach, taking into account various factors such as climatic conditions, plant phenology, infestation rates, and the rate of pest spread. This holistic perspective recognizes the intricate web of interactions within the ecosystem and acknowledges the multifaceted nature of mite dynamics.

In essence, the conclusion provides a foundation for future research endeavors to delve deeper into the complexities of mite–plant interactions, urging researchers to consider a broader set of variables for a more comprehensive understanding of these ecological dynamics.
